# Intracellular Delivery of Anti-SMC2 Antibodies against Cancer Stem Cells

**DOI:** 10.3390/pharmaceutics12020185

**Published:** 2020-02-21

**Authors:** Sara Montero, Joaquin Seras-Franzoso, Fernanda Andrade, Francesc Martinez-Trucharte, Mireia Vilar-Hernández, Manuel Quesada, Helena Xandri, Diego Arango, Ibane Abasolo, Diana Rafael, Simo Schwartz

**Affiliations:** 1 Drug Delivery and Targeting Group, Molecular Biology and Biochemistry Research Centre for Nanomedicine (CIBBIM-Nanomedicine), Vall d’Hebron Institut de Recerca, Universitat Autònoma de Barcelona, 08035 Barcelona, Spain; sara.montero@vhir.org (S.M.); joaquin.seras@vhir.org (J.S.-F.); fernanda.silva@vhir.org (F.A.); francesc.martinez@vhir.org (F.M.-T.); mireia.vilar@alumni.vhir.org (M.V.-H.); mbenque@correo.ugr.es (M.Q.); helena.xandri@alumni.vhir.org (H.X.); 2 Networking Research Centre for Bioengineering, Biomaterials, and Nanomedicine (CIBER-BBN), Instituto de Salud Carlos III, 28029 Madrid, Spain; 3 i3S—Instituto de Investigação e Inovação em Saúde, Universidade do Porto, Rua Alfredo Allen 208, 4200-180 Porto, Portugal; 4 INEB—Instituto Nacional de Engenharia Biomédica, Universidade do Porto, Rua Alfredo Allen 208, 4200-180 Porto, Portugal; 5 Biomedical Research in Digestive Tract Tumors, CIBBIM-Nanomedicine, Vall d’Hebron Institut de Recerca, Universitat Autonoma de Barcelona, 08035 Barcelona, Spain; diego.arango@vhir.org; 6 Immune Regulation and Immunotherapy, CIBBIM-Nanomedicine, Vall d’Hebron Institut de Recerca, Universitat Autonoma de Barcelona, 08035 Barcelona, Spain; 7 Functional Validation and Preclinical Research, Molecular Biology and Biochemistry Research Centre for Nanomedicine (CIBBIM-Nanomedicine), Vall d’Hebron Institut de Recerca, Universitat Autònoma de Barcelona, 08035 Barcelona, Spain

**Keywords:** SMC2, condensin complexes, nanomedicine, polymeric micelles, cancer stem cells, 5-FU, PTX, breast cancer, colon cancer, antibody intracellular delivery

## Abstract

Structural maintenance of chromosomes protein 2 (SMC2) is a central component of the condensin complex involved in DNA supercoiling, an essential process for embryonic stem cell survival. SMC2 over-expression has been related with tumorigenesis and cancer malignancy and its inhibition is regarded as a potential therapeutic strategy even though no drugs are currently available. Here, we propose to inhibit SMC2 by intracellular delivery of specific antibodies against the SMC2 protein. This strategy aims to reduce cancer malignancy by targeting cancer stem cells (CSC), the tumoral subpopulation responsible of tumor recurrence and metastasis. In order to prevent degradation and improve cellular internalization, anti-SMC2 antibodies (Ab-SMC2) were delivered by polymeric micelles (PM) based on Pluronic^®^ F127 amphiphilic polymers. Importantly, scaffolding the Ab-SMC2 onto nanoparticles allowed its cellular internalization and highly increased its efficacy in terms of cytotoxicity and inhibition of tumorsphere formation in MDA-MB-231 and HCT116 breast and colon cancer cell lines, respectively. Moreover, in the case of the HCT116 cell line G1, cell-cycle arrest was also observed. In contrast, no effects from free Ab-SMC2 were detected in any case. Further, combination therapy of anti-SMC2 micelles with paclitaxel (PTX) and 5-Fluorouracil (5-FU) was also explored. For this, PTX and 5-FU were respectively loaded into an anti-SMC2 decorated PM. The efficacy of both encapsulated drugs was higher than their free forms in both the HCT116 and MDA-MB-231 cell lines. Remarkably, micelles loaded with Ab-SMC2 and PTX showed the highest efficacy in terms of inhibition of tumorsphere formation in HCT116 cells. Accordingly, our data clearly suggest an effective intracellular release of antibodies targeting SMC2 in these cell models and, further, strong cytotoxicity against CSC, alone and in combined treatments with Standard-of-Care drugs.

## 1. Introduction

Protein therapy has emerged as a promising therapeutic alternative due to its high specificity and reduced off-target effects [1]. Among protein-based therapies, the use of antibodies have been largely explored and numerous treatments are currently available for a broad spectrum of clinical indications, from transplant rejection to cancer [2]. One of the most interesting features of antibody-based therapies is their potential to reach a wide range of targets, including those that are usually unattainable by other strategies (i.e., undruggable targets). However, because most antibodies are unable to easily cross the extracellular membrane, their applications are often limited to extracellular targets [3,4]. In the last years, several research groups have explored the use of antibodies against intracellular targets. Straightforward techniques for antibody delivery, such as microinjection, electroporation and antibody modification with cell penetration peptides (CPPs), have been tested. Unfortunately, even though experimentally effective in vitro, they have not been efficiently translated to in vivo models or into clinical applications [5,6]. In addition, although different nanoparticles, mainly metallic and lipidic, have been developed for intracellular delivery of antibodies, only few of them showed efficient intracellular delivery [7,8]. The current drawbacks are related with limited cargo for hydrophilic substances, un-adequate endosomal escape, in vivo instability and a short half-life circulation time. Thus, intracellular delivery of antibodies is still a considerable challenge [9,10].

On the other hand, although extensively investigated and besides constant development of new therapies, cancer often remains as an incurable disease. Insights into cancer physiology have shown the complexity of cancer regulatory networks in which a large variety of different cell types are involved and largely interconnected to determine disease progression. In this regard, particular attention should be addressed to metastatic spreading, resistance to treatment and tumor recurrence. These features are related with the presence of a cancer cell subpopulation known as cancer stem cells (CSC). CSC display stemness properties, such as self-renewal, quiescence and the capability to remain in an undifferentiated state [11,12]. Besides, CSC have been shown to exhibit high expression levels of drug efflux transporters and unregulated DNA repair machinery, conferring them drug resistance potential. In this context, CSC have become a highly appealing target in order to improve current anti-cancer therapies [13,14]. Although the number of new drugs with therapeutic potential against CSC is rising and several products are already in clinical trials, there is still strong need to broaden the catalogue of therapeutic options.

In this sense, the Structural Maintenance of Chromosomes 2 (SMC2) protein could be a good therapeutic target candidate. SMC2 forms part of the condensin complex that has a central role in many aspects of chromosome biology, including the segregation of sister chromatids and compaction of chromosomes during cell division, as well as the regulation of gene expression during the interphase [15,16,17,18]. Importantly, SMC2 protein has been mostly located at the cell cytoplasm during cell interphase with only a minor amount remaining associated to chromatin in the nucleus. The condensin complex is formed by five subunits and requires the initial arrangement of a SMC2/SMC4 heterodimer. The condensin complex is completed by the union of a subcomplex, formed by the three non-SMC subunits (Cnd1, Cnd2 and Cnd3), to the heterodimer of SMC2/SMC4 [15,16,17]. Of note, SMC2 has been found to be over-expressed in a significant number of patients with colorectal cancer, gastric cancer, lymphoma and some types of neuroblastoma [19,20], and has been suggested as a risk biomarker in pancreatic cancers [21]. Moreover, when SMC2 expression was knocked down it drastically reduced tumor growth in colorectal cancer mice models [18]. This data and the central role of the condensin complex in cell division suggested that an effective inhibition of the activity of this complex would prevent cancer cells from dividing. Under this premise, directing SMC2-targeted inhibitory molecules into the cell cytoplasm should ensure SMC2 protein blockade and serve as a novel therapeutic strategy.

Hereby we propose a polymeric micelle delivery system (PM) based on amphiphilic polymers, namely the Pluronic^®^ F127 as a nanoplatform to cluster the antibodies directed against the SMC2 protein. Because of its small size, biocompatibility, stealth properties and amphiphilic nature, this is an ideal system to overcome major drawbacks related to intracellular delivery of antibodies. Furthermore, poloxamer polymers like Pluronic^®^ F127 have shown to interfere with the function of P-glycoprotein, being therefore helpful to further overcome drug resistance and reduce cancer recurrence [22,23,24]. Moreover, taking advantage of the plasticity provided by amphiphilic PM we have also explored potential combinatorial therapeutic strategies with 5-FU and PTX, drugs commonly used in colon and breast cancer, respectively.

## 2. Materials and Methods

### 2.1. Cell Lines and Culture Conditions

The HCT116 (ATCC^®^ CCL-247™) colon cancer cell line, MDA-MB-231 (ATCC^®^ HTB-26™) breast cancer cell line and PANC-1 (ATCC^®^ CRL-1469™) pancreas cancer cell line were obtained from American Type Culture Collection (ATTC^®^, LGC Standards, Barcelona, Spain). Cells were cultured in RPMI medium (ThermoFisher Scientific, Madrid, Spain supplemented with 10% Fetal Bovine Serum (FBS) (ThermoFisher Scientific, Madrid, Spain), a 1% penicillin–streptomycin mixture (ThermoFisher Scientific, Madrid, Spain), 1% *L*-glutamine (Lonza, Basel, Switzerland) and 1% Non-Essential Amino Acids (NEAA) (ThermoFisher Scientific, Madrid, Spain). All cell lines were maintained at 37 °C under a 5% CO_2_-saturated atmosphere. The medium was changed every two days, and upon sub-confluence, cells were harvested from plates with 0.25% trypsin-EDTA and subcultured (ThermoFisher Scientific, Madrid, Spain).

Cells were also cultured in low attachment conditions as tumorspheres in serum-free media and seeded in ultra-low attachment surface plates (Corning Life Sciences, Chorges, France). The medium for breast and pancreas sphere growth was supplemented with glucose, 60 mg/mL (Merck Life Science S.L.U., Madrid, Spain), as well as 10 µL/mL L-Glutamax (Merck Life Science S.L.U., Madrid, Spain), 10 µL/mL antibiotic–antimitotic mixture (Merck Life Science S.L.U., Madrid, Spain), 4 µg/mL heparin (Merck Life Science S.L.U., Madrid, Spain), 2 mg/mL BSA (Merck Life Science S.L.U., Madrid, Spain), 0.02 µg/mL of human recombinant EGF (Merck Life Science S.L.U., Madrid, Spain), 0.01 µg/mL of human recombinant bFGF (ThermoFisher Scientific, Madrid, Spain) and 10% of Hormone Mix: 10 µg/mL putrescin, 20 µM progesterone (both from Merck Life Science S.L.U., Madrid, Spain), 0.1 mg/mL apo-transferrin, 25 µg/mL insulin and 30 µM selen (ThermoFisher Scientific, Madrid, Spain). Tumorspheres from the HCT116 cell line were also cultured in serum-free media supplemented with a 1% antibiotic–antimitotic mixture, 10 ng/mL of human recombinant EGF, 20 ng/mL of human recombinant bFGF and 10 ng/mL of LIF Recombinant Human Protein (TermoFisher Scientific, Madrid, Spain).

### 2.2. Phase Contrast Microscopy

The morphology and structure of adherent cells and spheres were routinely assessed by phase contrast microscopy imaging after treatment with the distinct compounds. Images were acquired at 10× amplification using a Nikon Eclipse TS100 (Nikon Instruments Europe BV, Amsterdam, Netherlands) inverted microscope and further edited with ImageJ 1.49v software.

### 2.3. Cell Transfection with siRNA

#### 2.3.1. Gene Expression Determination

10^5^ cells were seeded in 6-well plates and transfected with SMC2-siRNA (SI02654260, Qiagen, Hilden, Germany) using Lipofectamine^®^ 2000 (ThermoFisher Scientific, Madrid, Spain) according to the manufacturer’s instructions. A nonspecific sequence was used as the control (siControl). The culture medium was changed 6 h after transfection and cells harvested after 72 h of incubation. Samples were further processed for RNA extraction as detailed below in Section 2.4.

#### 2.3.2. Cell Growth Assay

After cell transfection, cells were washed in PBS and harvested by trypsin digestion following standard procedures. Cells were counted in a Countess^TM^ II (ThermoFisher Scientific, Madrid, Spain).

#### 2.3.3. Sphere Formation Assay

SMC2 knock-down was performed following a 2-times siRNA shot strategy in order to guarantee an efficient gene expression inhibition along the assay. Briefly, a first treatment with SMC2-siRNA using Lipofectamine^®^ 2000 (ThermoFisher Scientific, Madrid, Spain) was performed as described above. After 72 h an identical SMC-siRNA shot was performed. After 6 h upon the second siRNA transfection, cells were harvested and washed 3 times in serum free media. Then, 1000 cells were seeded in ultra-low attachment 96-well plates, adding sphere growth media for assessing sphere formation (as described in the cell viability assay Section 2.10.1).

### 2.4. RNA Extraction and Real-Time Quantitative Reverse Transcription Polymerase Chain Reaction (qRT-PCR)

Total RNA was extracted from cells using RNeasy Micro Kit (Qiagen, Hilden, Germany), and the obtained RNA was reverse transcribed with a High Capacity cDNA Reverse Transcription Kit (Applied Biosystems, Madrid, Spain), according to the manufacturer’s instructions. The cDNA reverse transcription product was amplified with specific primers for SMC2 (hSMC2 F: 5′ AAT GAG CTG CGG GCT CTA GA 3′; hSMC2 R: 5′ TTG TTG CTT GTG ATA TGA GCT TTG 3′); GADPH (hGADPH F: 5′ ACC CAC TCC TCC ACC TTT GAC; hGADPH R: 5′ CAT ACC AGG AAA TGA GCT TGA CAA 3′) and Actin (hActin F: 5′ CAT CCA CGA AAC TAC CTT CAA CTC C 3′; hActin R: 5′GAG CCG CCG ATC CAC AC 3′) by qPCR using SYBR Green (ThermoFisher Scientific, Madrid, Spain) to fluorescently label double stranded DNA. The reaction was performed on a 7500 Real time PCR system (Applied Biosystems, Madrid, Spain). At least three biological replicates, each comprising two technical replicates, were performed. Relative normalized quantities (NRQ) of mRNA expression were calculated using the comparative Ct method (2e-ΔΔCt) with two reference genes (hGAPDH and hActin) used as endogenous controls through Qbase™ software v3.2.

### 2.5. Protein Extraction and Western Blotting

Cell pellets were lysed with the Cell Lytic M reagent (Merck Life Science S.L.U., Madrid, Spain) containing a protease inhibitor cocktail (Roche cOmplete™, Merck Life Science S.L.U., Madrid, Spain). Proteins in the crude lysates were quantified using the Pierce^TM^ BCA protein quantification kit (ThermoFisher Scientific, Madrid, Spain) following the manufacturer’s instructions. A total of 20 μg of whole-cell lysates were separated by SDS-PAGE and transferred onto PVDF membranes. Blots were probed using primary antibodies anti-SMC2 (Merck Life Science S.L.U., Madrid, Spain) and *β*-Tubulin (Invitrogen, ThermoFisher Scientific, Madrid, Spain). Proteins were detected using HRP-conjugated secondary antibodies (Dako, Palex Medical SA, Barcelona, Spain) and developed after appropriate incubation in an Immobilion^®^ Western reagent (Merck Life Science S.L.U., Madrid, Spain) using an Odissey FC imaging system (LI-COR Biotechnology GmbH, Bad Homburg, Germany) for chemiluminescence detection.

### 2.6. Production of Polymeric Micelles

Pluronic^®^ F127 was kindly provided by BASF (Ludwigshafen, Germany). PM were prepared using the thin-film hydration technique [22]. Pluronic^®^ F127 carboxylation was performed as previously reported [25]. Briefly, F127 and F127:COOH polymers were individually weighed in an 8:2 (*w*/*w*) ratio and dissolved in a mixture of methanol:ethanol (1:1) (Merck Life Science S.L.U., Madrid, Spain). For the formation of loaded micelles, the drug, 5-FU or PTX (both from Merck Life Science S.L.U., Madrid, Spain) were added to the organic solution at the desired concentration. Then, the solvent was removed under vacuum in a rotary evaporator (bath temperature 60 °C, 200 rpm), and the resulting film left to dry overnight at room temperature to eliminate any remaining solvent. The film was then hydrated with PBS and vortexed for 5 min at full speed. For the PM encapsulating SMC2 antibodies (PM:SMC2), the antibody (Anti-SMC2/hCAP-E Antibody, Merck Life Science S.L.U., Madrid, Spain) was added at the aqueous phase during the rehydration step. For PM functionalized with SMC2 antibodies (PM-CON:SMC2), an adequate amount of EDC (polymer:EDC ratio 1:1.5) (Merck Life Science S.L.U., Madrid, Spain) was incubated with the formulation during 30 min at RT. Afterwards, the SMC2 antibody solution was added and incubated under stirring during 2 h at RT. Samples were freeze-dried for long-term storage using a VirTisBenchTop Freeze-Dryer (SP Scientific, Ipswich, UK) when required.

### 2.7. Physicochemical Characterization of the Polymeric Micelles

#### 2.7.1. Zeta Potential Measurements

Zeta potential was assessed by laser Doppler microelectrophoresis using a NanoZS (Malvern Instruments, Malvern, UK) with an angle of 173°. Samples were diluted 1:5 in Milli-Q^®^ water (18.2 MΩ·cm at 25 °C) in order to obtain an adequate nanoparticle concentration. Data obtained from each formulation were represented as mean values and measured at least in triplicate.

#### 2.7.2. Transmission Electron Microscopy (TEM)

Particle shape and morphology were observed by TEM, using a JEM-1400 Electron Microscope (JEOL Ltd., Croissy-sur-Seine, France) with an applied voltage of 120 kV. For that, a drop of sample (previously diluted 1:20 in Milli-Q^®^ water) was placed on a carbon 400-mesh copper grid, the liquid excess removed, and the sample contrasted with uranyl acetate before visualization. Image J 1.49v software was used to process information and determine diameter measures (*n* > 200) from TEM images, while histogram plots from nanoparticles size distribution were generated by GraphPad Prism 6. The dispersion index (d) was determined by Equation (1).

Standard Deviation (SD)/Particle Size Arithmetic Mean(1)

#### 2.7.3. Loading/Association Efficiency Determination

The efficacy of SMC2 loading in the case of PM:SMC2 and association efficiency in the case of PM-CON:SMC2 was assessed by BCA protein assay. Briefly, the amount of free SMC2 antibody in the aqueous phase of the PM was separated by centrifugation with filtration (10,000 *g*, 10 min at RT) using 300k membrane centrifugal devices (Nanosep^®^ Centrifugal Devices, Pall España, Madrid, Spain). Then, the protein level was measured from the flow using a Pierce^TM^ BCA protein assay kit (ThermoFisher Scientific, Madrid, Spain). The Loading/Association efficiency percentage was determined according to Equation (2):

Total amount of SMC2 (unfiltered PM) − free SMC2 in filtrate/Total amount of SMC2 (unfiltered PM) × 100(2)

#### 2.7.4. Fourier-Transform Infrared Spectroscopy (FTIR)

The conjugation of the SMC2 antibody at the PM surface was also confirmed by FTIR analysis. FTIR was carried out using a spectrometer Perkin-Elmer Spectrum One (energy range: 450–4000 cm^−1^) equipped with a Universal Attenuated Total Reflectance Accessory (U-ATR, Perkin-Elmer, Madrid, Spain). Prior to analysis, samples were freeze-dried using a VirTisBenchTop Freeze-Dryer (SP Scientific, Ipswich, UK).

### 2.8. Micelles Internalization: Flow Cytometry and Confocal Microscopy

Cell internalization of the different formulations was assessed in HCT116 and MDA-MB-231 using 5-DTAF (Merck Life Science S.L.U., Madrid, Spain) fluorescently labeled PM [25,26].

#### 2.8.1. Flow Cytometry

Briefly, 20,000 cells were seeded in complete RPMI medium in 96-well plates and left to attach for 24 h. PM were added to cells (10 mg/mL) and incubated for 15, 30, 60, 180, 360 and 1440 min, respectively. Then, cells were washed with 1× PBS, trypzinized and neutralized with PBS supplemented with 10% FBS and 1 μg/mL DAPI (Merck Life Science S.L.U., Madrid, Spain) used for vital staining. The plate was read in a cytometer Fortessa (BD Biosciences, Madrid, Spain) and data was analyzed with FCS Express 4 Flow Research Edition software (De Novo Software, Los Angeles, USA, Version 4). Three biological replicated were performed for each condition and only DAPI negative cells were admitted for the analysis, while cell debris or possible aggregates were removed by forward and side scatter gating. For each sample, at least 10,000 individual cells were collected, and the percentage of fluorescent cells evaluated.

#### 2.8.2. Confocal Microscopy

A total of 50,000 cells were seeded in complete RPMI medium in 8-well chambered coverglass (ThermoFisher Scientific, Madrid, Spain) and incubated overnight at 37 °C and 5% CO_2_ to allow cell adhesion. Cells were incubated with 5-DTAF-fluorescently labelled PM (10 mg/mL) for 6 h. Lysosomes were stained with 1 µM Lysotracker^®^ Red DND-99 (ThermoFisher Scientific Madrid, Spain) for 30 min at 37 °C while the cell membrane was stained with 5 µg/mL CellMask™ (Invitrogen, ThermoFisher Scientific, Madrid, Spain) for 15 min at 37 °C. Subsequently, cells were fixed in 4% PFA (Merck Life Science S.L.U., Madrid, Spain) at 4 °C for 20 min followed by nuclei staining with DAPI (1 μg/mL) for 5 min at RT in the dark. Cells were viewed under a Spectral Confocal Microscope MFV1000 Olympus (Olympus Iberia, S.A.U., L’Hospitalet de Llobregat, Spain). The 561 nm excitation wavelength of the green laser (10 mW) was used for selective detection of the red fluorochromes (Lysotracker^®^ Red and CellMask™). The 488 nm excitation wavelength of Argon multiline laser (40 mW) was used for selective detection of the green fluorochrome (5-DTAF). The nuclear staining DAPI was excited at 405 nm with a violet laser (6 mW). Minimal single optical sections were collected for each fluorochrome sequentially. Images were merged and analyzed with Image J 1.49v software.

### 2.9. Cell Cycle Assay by Flow Cytometry

A flow cytometric analysis of cell cycle with propidium iodide DNA staining was carried out. Briefly, 40,000 cells of HCT116 cells and 30,000 cells of MDA-MB-231 cells were seeded in 24-well plates and left to adhere during 24 h at 37 °C and a 5% CO_2_-saturated atmosphere. Then, cells were treated and incubated with empty PM (control PM) (5 mg/mL) and PM-CON:SMC2 (5 mg/mL polymer and 32.9 µg/mL of SMC2 antibody) for 48 h. Untreated cells were used as the negative control. Afterwards, cells were harvested, centrifuged (8000 rpm, 5 min at 4 °C) and fixed in cold 70% ethanol for 30 min at 4 °C. After centrifugation, cells were washed two times with PBS and the cell pellets resuspended in 250 µL of the staining solution containing 100 µg/mL of ribonuclease PureLink™ RNase A (ThermoFisher Scientific, Madrid, Spain) and 50 µg/mL of propidium iodide (Merck Life Science S.L.U., Madrid, Spain) in order to ensure that only DNA, not RNA, was stained.

Cell cycle evaluation was performed using an FACS Calibur (BD Biosciences, Madrid, Spain) flow cytometer and the resulting histograms analysed with FCS Express 4 Flow Research Edition software (De Novo Software, Version 4). Three biological replicates were performed and at least 5000 individual cells were collected for the analysis once debris were removed by forward and side scatter gating. The multicycle option in autofit mode was performed in order to automatically quantify the percentage of cells in each cell cycle phase. By this method a Gaussian curve was fitted to each phase allowing the precise determination of the area under the curve. In addition, the G2/G1 ratio was also represented, scoring around 2 in all the cases, in compliance with the accepted quality standards for cell-cycle assays.

### 2.10. Tumor Cell Viability Assays

#### 2.10.1. Viability Assay in Adherent Cell Cultures

3000 cells of the HCT116 cell line and 5000 cells of the MDA-MB-231 cell line were seeded in 96-well plates and incubated in overnight to allow adhesion. Then, cells were treated and incubated during 72 h with crescent concentrations of the different formulations (PM, PM:SMC2 and PM-CON:SMC2) at 5 mg/mL of polymer and 32.9 µg/mL of SMC2 antibody. To determine the cytotoxicity of free 5-FU and free PTX (Merck Life Science S.L.U., Madrid, Spain), cells were incubated during 48 h with a range concentration of 5-FU and PTX of 384.40 µM to 0.023 µM and 1 µM to 0.0078 µM, respectively. Complete medium was used as negative control and 10% DMSO as positive control of toxicity. Cell viability was measured using the 3-(4,5-dimethylthiazol-2-yl)-2,5-diphenyltetrazolium bromide (MTT) reagent (Merck Life Science S.L.U., Madrid, Spain). The absorbance of each well was read on an absorbance microplate reader ELx800 (BioTek, Colmar, France), at 590 and 630 nm for 5-FU and PTX, respectively. The half-maximal inhibitory concentration (IC_50_) was determined by nonlinear regression of the concentration-effect curve fit using Prism 6.02 software (GraphPad Software, Inc.).

#### 2.10.2. Tumorsphere Viability Assays

Studies were performed using ultra-low attachment surface plates (Corning Life Sciences, Chorges, France). Cells were cultured in serum free media supplemented differently for HCT116 colon cell line and MDA-MB-231 breast cancer cell line, as mentioned above. HCT116, MDA-MB-231 and Panc-1 cells were transfected with siRNA anti-SMC2, as previously described. A total of 1000 cells were seeded and cultured for 6 days in ultra-low attachment plates.

For PM and drug efficacy assays, 1000 cells from the HCT116 cell line and 2000 cells from the MDA-MB-231 cell line were seeded in 96-well ultra-low attachment plates in serum-free media, supplemented as described above. After overnight incubation, cells were treated with different PM formulations (PM, PM-CON:SMC2, PM/5-FU, PM-PTX, PM-CON:SMC2/5-FU, PM-CON:SMC2/PTX) (1 mg/mL of polymer), the free drugs (5-FU, 3.84 µM and PTX, 0.1 µM) and free SMC2 antibody (6.58 µg/mL) for 7 days. The IC_50_ of the free tested drugs were also determined in low attachment conditions. Cells were treated with serial 1:2 dilutions of 5-FU (from 768.80 µM to 0.047 µM) and PTX (from 10 µM to 0.078 µM) for 7 days. Free serum medium was used as negative control and 10% DMSO as positive control of toxicity. In all cases, sphere formation was monitored with a Nikon Eclipse TS100 inverted microscope (Nikon Instruments Europe BV, Amsterdam, Netherlands) and quantified by Presto Blue^®^ Reagent assay (ThermoFisher Scientifics, Madrid, Spain). The absorbance of each well was read on a Multiskan^TM^ FC Microplate Photomer (ThermoFisher Scientific, Madrid, Spain), at 570 and 620 nm, and the data were processed by GraphPad Prism 6 software.

### 2.11. Statistical Analysis

At least three batches of each PM were produced and characterized. The results were expressed as the mean ± standard deviation. For biological studies, at least 3 replicates, each involving at least two technical replicates, were involved in the final results expressed as the mean ± standard deviation. Statistical analysis was performed in GraphPad Prism 6 software using a non-parametric Dunn test for multiple comparisons and Mann–Whitney U test for simple comparisons. Differences were regarded as statistically significant when the *p*-value was smaller than 0.05.

## 3. Results

### 3.1. SMC2 Silencing Causes Strong Reduction of Tumorsphere Formation in Cancer Cells

**Figure 1:**
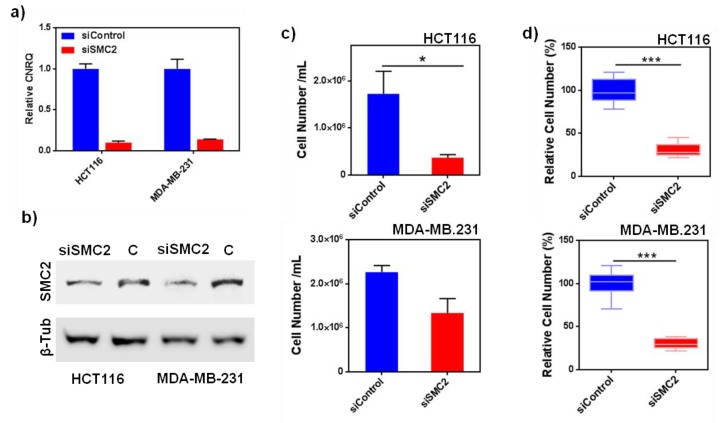
SMC2 siRNA inhibition. (**a**) Relative mRNA levels after SMC2 silencing for HCT116 and MDA-MB231 cell lines (siSMC2). Fold change is represented with respect to mRNA levels obtained from non-relevant siRNA-treated cells (siControl); (**b**) SMC2 and beta tubulin proteins detected by Western blot after SMC2 silencing (siSMC2) and compared to non-relevant siRNA-treated cells C; (**c**) cell viability assay for HCT116 and MDA-MB-231 adherent cells after treatment with siRNA against SMC2 (siSMC2) and with a scrambled control siRNA (siControl); and (**d**) cell viability assay for HCT116 and MDA-MB-231 growing in non-adherent conditions as tumorspheres, after treatment with siRNA against SMC2 (siSMC2) and with a scramble control siRNA (siControl). * *p* < 0.05, *** *p* < 0.001.

SMC2 siRNA efficiently reduces the expression of the gene at mRNA and protein level in the three different cell lines. Namely, colorectal cancer cell line HCT116, triple negative breast cancer cell line MDA-MB-231 and pancreatic cancer cell line PANC-1 (Figure 1a,b and Figure S1a,b). Interestingly, the effect of SMC2 silencing resulted in a clear decrease in cell viability in HCT116, whereas only a trend to reduce the number of cells was observed for MDA-MB-231 (Figure 1c); no difference could be detected in other tumor types tested as in the case of PANC-1 (Figure S1c). Importantly, when cells were serum deprived and forced to grow in absence of cell-surface contacts, a remarkable decrease of tumorspheres proliferation was observed in all cell lines treated with SMC2 siRNA (Figure 1d and Figure S1d). Of note, as previously reported by our group and others [27,28], cells growing as spheres display CSC-like features (i.e., HCT116 cell line; Figure S2).

### 3.2. Physicochemical Characterization of Polymeric Micelles with Conjugated or Encapsulated SMC2 Antibodies

**Figure 2:**
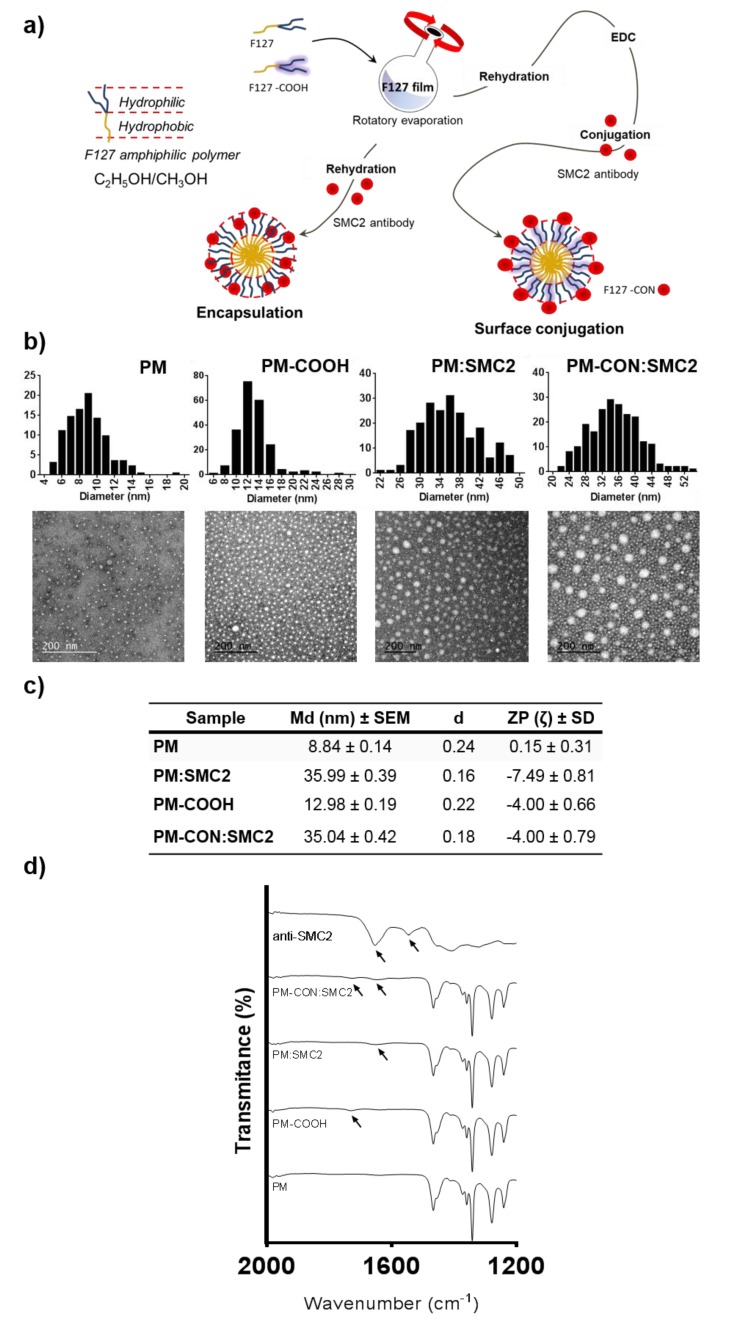
PM-Ab-SMC2 characterization. (**a**) Schematic representation of the two strategies employed for PM synthesis loaded with anti SMC2 antibody, namely encapsulation and surface conjugation. (**b**) TEM micrographs of the four distinct PM formulations. The panels above the micrographs correspond to the size distribution of the particles obtained by TEM image analysis. Scale bar represents 200 nm. (**c**) Summary table of mean diameter (Md), Dispersity Index (**d**), Zeta Potential (ZP) and (**d**) FTIR spectra, displaying the appearance of a peak at 1646 cm^−1^ and corresponding to amide I bond formation.

In order to develop a drug delivery system able to target SMC2 protein intracellularly, anti-SMC2 antibodies (Ab-SMC2) were successfully conjugated onto PM using two different approaches: (1) encapsulation by affinity into the PM hydrophilic shell (PM:SMC2) and (2) by covalent conjugation between the –COOH terminals of the PM and the -NH2 groups present in Ab-SMC2 *(PM-CON:SMC2)*. Particles TEM analysis revealed spherical morphologies in all cases and displayed rather homogenous populations in size (Figure 2b). The PM obtained by the first approach presented a mean diameter of 35.99 ± 0.39 nm with a dispersity index of 0.16, and a slightly negative superficial charge measured by NanoZS (Figure 2c). The micelles produced according to the second approach presented a mean diameter of (35.04 ± 0.42 nm), a d value of 0.18 and are faintly negatively charged (Figure 2c).

Regarding the antibody loading/association efficiency, it was determined by the measurement of free antibody in the micelle’s supernatant after 300 KDa filtration. PM:SMC2 presented an SMC2 loading efficiency of 68.02% ± 1.01% and PM-CON:SMC2 showed an association efficiency of 61.49% ± 3.11%. Moreover, the loading/association of Ab-SMC2 at the micelles surface were confirmed by FTIR (Figure 2d). Regarding FTIR analysis, control PM-COOH showed a peak at 1727 cm^−1^ corresponding to the –C=O stretching of the –COOH groups of the carboxylated Pluronic^®^ F127. As expected, this peak is absent in control PM composed only by unmodified Pluronic^®^ F127 [25]. Ab-SMC2 showed a peak at 1654 cm^−1^ corresponding to the C=O stretching vibrations of the peptide bond of the amide I and also a peak at 1545 cm^−1^ related to the N–H bending vibration/C–N stretching vibration of the amide II [29].

In the case of PM:SMC2, it was possible to observe a small shift of the peak corresponding to the amide I of Ab-SMC2 from 1654 to 1648 cm^−1^, possibly due to non-covalent interactions/hydrogen bonding between the protein and the Pluronic^®^ F127 [30]. In the case of PM-CON:SMC2, a small shift together with an increase in the intensity of the peak of the amide I (1646 cm^−1^) were detected, probably due to the conjugation of the –NH2 of Ab-SMC2 with the –COOH from carboxylated Pluronic^®^ F127 and the formation of new bonds. A small peak shift (1726 cm^−1^) accompanied by a drop of intensity of the –COOH peak also suggested a reduction of the availability of these free groups due to conjugation with –NH2. Altogether, these results suggest a covalent conjugation of Ab-SMC2 to the surface of the PM. In order to better confirm the peaks, PM-CON:SMC2 were produced with 100% of carboxylated Pluronic^®^ F127 (Figure S3). A peak at 1728 cm^−1^, corresponding to the –C=O stretching of the –COOH groups, and a peak at 1653 cm^−1^, corresponding to the C=O stretching vibrations of the peptide bond of the amide I, were observed. The lower shift of the amide I peak could be a result of the absence of the –OH groups from the unmodified Pluronic^®^ F127 and, consequently, lower hydrogen bonding interactions.

### 3.3. In Vitro Efficacy of PM-SMC2 and PM-CON:SMC2 in Adherent Cultures

**Figure 3:**
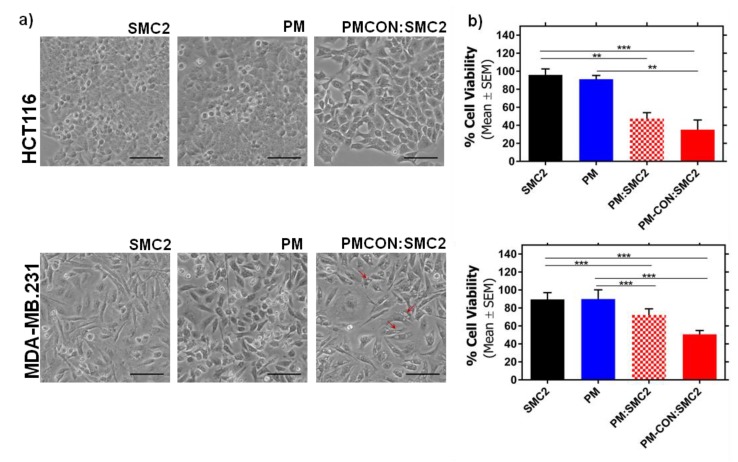
In vitro efficacy of Ab-SMC2 in tumor cell lines. (**a**) Phase contrast images of HCT116 (above panel) and MDA-MB-231 (below) after 48 h incubation with free SMC2 antibody, control PM and PM-CON:SMC2. Red arrows point at vacuoles appeared after PM-CON:SMC2 treatment. Scale bar correspond to 100 µm; (**b**) cell viability representation of HCT116 (above panel) and MDA-MB-231 (below) after 72 h of incubation with anti SMC2 antibody, control PM, PM:SMC2 and PM-CON:SMC2. The concentrations of the antibody and Pluronic^®^ 127 were 32.9 µg/mL and 5 mg/mL, respectively. These concentrations were also maintained in both formulations: PM:SMC2 and PM-CON:SMC2. ** *p* < 0.01, *** *p* < 0.001.

Cell viability assays were performed in HCT116 colon and MDA-MB-231 breast cancer cells. PM-SMC2 and PM-CON:SMC2 formulations were compared with the free SMC2 antibody and control PM. As expected, control PM and the free antibody did not show cell cytotoxicity at the maximum tested dose, namely 32.9 µg/mL for Ab-SMC2 and 5 mg/mL for Pluronic^®^ 127 (Figure 3a,b). On the other hand, the two formulations presented a significant cytotoxic effect in both cell lines at 72 h of incubation (Figure 3b). Nonetheless, PM-CON:SMC2 displayed a slight advantage in terms of effectiveness comparing to PM:SMC2. Thus, we decided to continue with the conjugated formulation of Ab-SMC2.

Further, we analyzed whether PM-CON:SMC2 might also cause changes in cell morphology and cell distribution in HCT116 and MDA-MB-231 models. Our data show a dramatic change in cell morphology in HCT116 cells. Cells treated with PM-CON:SMC2 showed a highly stretched shape and formed significantly less cell clusters than free Ab-SMC2 and empty PM (control PM). For fibroblast-shaped MDA-MB-231 cultures, cells treated with PM-CON:SMC2 displayed similar morphology and distribution than controls. Interestingly, a significant number of vacuoles were observed in samples incubated with PM-CON:SMC2 whereas no such structures were detected with free Ab-SMC2 and control PM (Figure 3a). These results show a biological activity of Ab-SMC2 when administered in PM that is not observed when PM are not employed.

### 3.4. PM-CON:SMC2 Micelles Show Faster Cellular Uptake than Control PM

**Figure 4:**
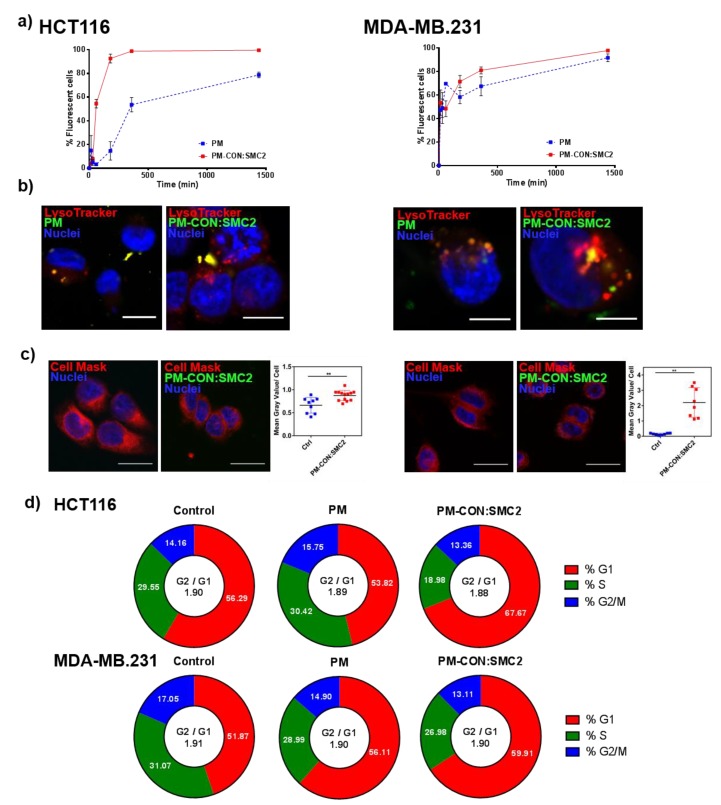
**** PM-CON:SMC2 uptake and intracellular fate. (**a**) Flow cytometry graphs displaying the percentage of fluorescent cells after HCT116 and MDA-MB-231 cell incubation with 5 mg/mL PM, PM:SMC2 and PM-CON:SMC2. (**b**) Confocal images showing either PM or PM-CON:SMC2 in green, acidic vesicles in red and nuclei in blue for HCT116 and MDA-MB-231 cells after 6 h incubation with 5 mg/mL PM. Scale bar represent 10 µm. (**c**) Confocal images displaying PM-CON:SMC2 in green, plasma membrane in red and nuclei in blue, for HCT116 and MDA-MB-231 cells after 6 h incubation with 5 mg/mL PM. Scale bar represent 20 µm. Side panels, graphical representations of green fluorescence measures in the cytoplasm. (**d**) Diagrams of cell cycle assay performed for HCT116 and MDA-MB-231 cells after 48 h of incubation with 5 mg/mL PM, PM-CON:SMC2 (32.9 µg/mL of antibody) and their respective untreated control. Percentages of cells at distinct cell cycle phases: G1, S and G2/M are displayed. The G2/G1 ratio is shown inside the circle. ** *p* < 0.01.

Cellular internalization and intracellular localization assessment of PM decorated with Ab-SMC2 were carried out at several time-points by flow cytometry. Accordingly, 5-DTAF fluorescently labeled PM-CON:SMC2 were incubated with HCT116 and MDA-MB-231 cells. Figure 4a shows that the conjugated nanoparticle (PM-CON:SMC2) presented a faster uptake profile than PM in both cell lines. Further, we could also observe that the MDA-MB-231 cell line exhibited faster uptake profiles than HCT116 cells, which indicates that internalization efficiency is largely dependent on the cell type.

Fluorescently labelled PM were also employed for confocal analysis, after 6 h of incubation with HCT116 and MDA-MB-231 cells. Acidic vesicles were labelled with Lysotracker^®^ Red to discriminate whether particles ended up into the lysosomes or were able to escape, at least partially, from the endosomal compartments. For all the tested formulations, we observed yellow dots corresponding to the merge of the green signal provided by DTAF and the red signal produced by the Lysotracker^®^ labelling late endosomes, lysosomes and other membrane structures with an acidic pH (Figure 4b). These results indicate that at least part of the formulated PM was endocyted and finally processed in the lysosomes. Nevertheless, because an effective blockage of SMC2 will require a cytosolic delivery of Ab-SMC2, we also measured the green fluorescence intensity at the cytoplasm of the HCT116 and MDA-MB-231 cells after their incubation with PM-CON:SMC2. For this, we used cell mask membrane staining and DAPI nucleus labelling to define a region of interest, namely the cellular cytosol. Excluding from the analysis bright green dots as PM accumulation within endosomal vesicles, we could detect an increase of green fluorescence in the cell cytoplasm of cells treated with PM-CON:SMC2, suggesting that this formulation was able to reach the cell cytoplasm (Figure 4c).

### 3.5. G1/S Cell Cycle Arrest Induced by PM-CON:SMC2

Finally, to further validate these results, we performed cell cycle assays in HCT116 and MDA-MB-231 cells treated with PM and PM-CON:SMC2. Of note, an effective targeting of intracellular SMC2 should have an impact in the cell cycle and reduce the number of cells into G2/M phase. As it can be observed in Figure 4d, HCT116 cells treated with PM-CON:SMC2 displayed a markedly higher percentage of cells in G1 in comparison to untreated cells and cells incubated with PM as controls. In addition, the percentage of cells in G2/M was also reduced in the PM-CON:SMC2-treated samples. For MDA-MB-231 the response pattern was similar when comparing untreated and PM-CON:SMC2-treated cells, exhibiting even higher differences regarding the decrease of G2/M percentage. Unexpectedly, control PM also showed an impact in cell-cycle arrest at G1 in this cell line (see Figure S3 for cell-cycle curve fitting examples).

### 3.6. Strong Anti-CSC Efficacy of PM-CON:SMC2 in Comparison with Free Ab-SMC2

**Figure 5:**
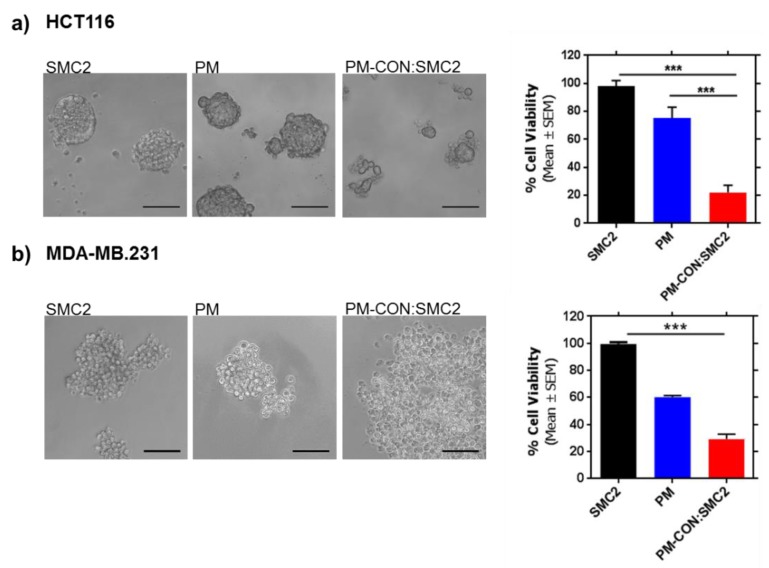
PM-CON:SMC2 in vitro efficacy in CSC. Left panels, microscopy images of HCT116 (**a**) and MDA-MB-231 (**b**) tumorspheres after treatment with Ab-SMC2 (6.58 µg/mL), control PM (1 mg/mL of Pluronic^®^ 127) and PM-CON:SMC2 (6.58 µg/mL of antibody and 1 mg/mL of Pluronic^®^ 127). Scale bar correspond to 100 µm. Right panels represent cell viability assays in HCT116 and MDA-MB-231 spheres growing for 7 days in non-adherent conditions. *** *p* < 0.001.

CSC growing under serum starvation conditions and in independent cell anchorage were employed. Tumorsphere formation assays were performed using HCT116 and MDA-MB-231 cancer cells treated with PM-CON:SMC2, Ab-SMC2 and control PM. As expected, free Ab-SMC2 did not produce any cytotoxic effect in sphere formation in any of the tested cell lines. Remarkably, strong cytotoxicity was observed when cells were treated with PM-CON:SMC2 for both cell lines, while control PM showed a slight decrease on the capacity of cells to produce tumorspheres. Furthermore, a deeper analysis of the structure of the tumorspheres showed that in PM-CON:SMC2 treated cells spheres shrank and displayed looser conformations, confirming the effects of PM-CON:SMC2 over CSC. These results suggest that SMC2 blockage is crucial in order to eliminate CSC. Moreover, these data confirm the intracellular delivery of Ab-SMC2 through PM also in a tumorsphere model (Figure 5).

### 3.7. PM-CON:SMC2/5-FU and PM-CON:SMC2/PTX Combined Effect

**Figure 6:**
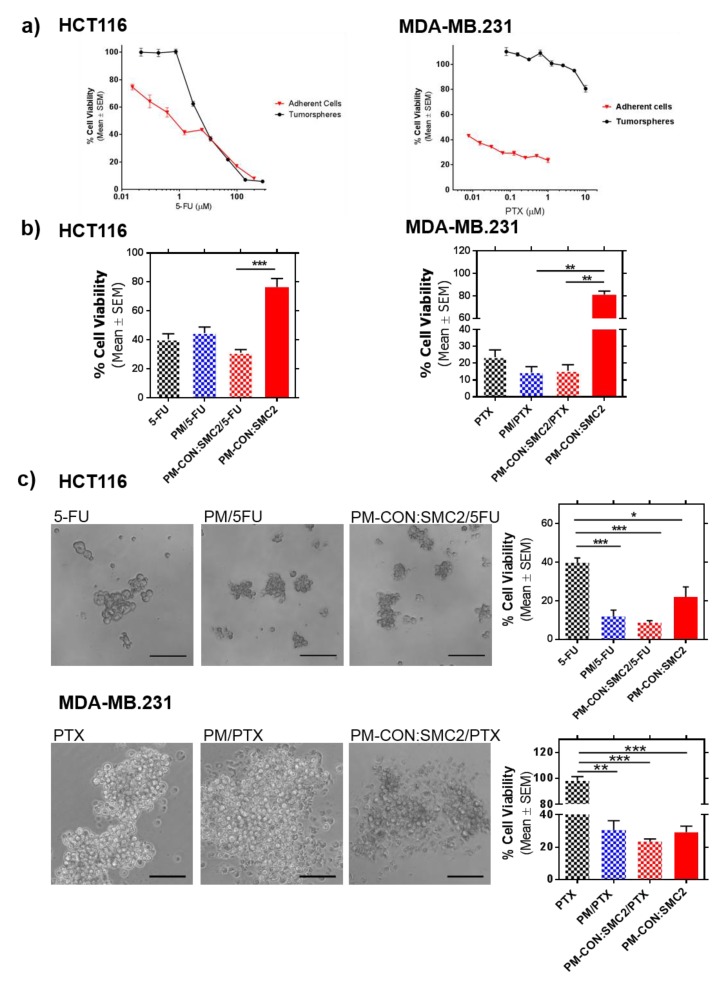
PM-Ab-SMC2/Drug combination. (**a**) Cell viability curves of HCT116 and MDA-MB-231 cells cultured in adherent culture or as tumorspheres after the incubation of increasing concentrations of 5-FU for HCT116 (from 0.023 µM to 384.40 µM for adherent conditions and from 0.047 µM to 768.86 µM for low-attachment conditions) and PTX for MDA-MB-231 cells (from 0.0078 µM to 1 µM for adherent growing cells and from 0.078 µM to 10 µM for tumorspheres). (**b**) Efficacy on adherent cells of the free drug (48 µM of 5-FU and 1 µM of PTX), encapsulated drug into PM (0.625 mg/mL for HCT116 and 5 mg/mL for MDA-MB-231 of polymer) and encapsulated drug into PM-CON:SMC2 (4.11 µg/mL for HCT116 and 32.9 µg/mL for MDA-MB-231 of Ab-SMC2). (**c**) Left panels, microscopy images of HCT116 and MDA-MB-231 spheres after treatment with the free drug (3.84 µM of 5-FU and 0.1 µM of PTX), PM/Drug (1 mg/mL of polymer) and PM-CON:SMC2/Drug (6.58 µg/mL of anti SMC2 antibody). Scale bar correspond to 100 µm. Right panels, sphere formation assay of HCT116 and MDA-MB-231 cells after treatment with the free drug, PM/Drug and PM-CON:SMC2/Drug. * *p* < 0.05, ** *p* < 0.01, *** *p* < 0.001.

In the clinical setting, PTX and 5-FU are often used in combined chemotherapeutic regimens against breast and colon cancer, respectively. Because a combination of different chemo-therapeutic drugs is commonly endured to improve clinical outcomes, we tested the cytotoxic activity of PM-CON:SMC2 loaded with either PTX or 5-FU anti-tumor drugs for combined treatments. For this, we took advantage of the amphiphilic nature of Pluronic^®^ 127. Thus, while Ab-SMC2 remained conjugated onto the PM surface, we were able to encapsulate these drugs into the hydrophobic core of the resulting PM. Interestingly, when looking respectively at the efficacy of 5-FU and PTX as free drugs in regular cell cultures of HCT116 and MDA-MB-231 in comparison to cells growing as tumorspheres (non-attachment), we observed that both drugs were significantly more active against adherent cells than against tumorspheres (Figure 6a). Tumorsphere resistance was noticeable for 5-FU in HCT116 at concentrations up to 1 µM. In the case of MDA-MB-231, tumorspheres showed resistance to PTX at all tested concentrations, including the highest one (10 µM) (Figure 6a, right panel). These results are in accordance to the CSC-like phenotype associated with tumorsphere growth and more specifically to the described drug resistance capacity of CSC.

Because eradicating CSC in tumors is becoming a great challenge for designing new treatments, we focused in the potential synergism of delivering Ab-SMC2 together with either PTX or 5-FU against tumor cells in non-adherent conditions. In this regard, the efficacies of 5-FU and PTX were tested at concentrations of 48 µM of 5-FU and 1 µM of PTX in HCT116 and MDA-MB-231 cells in adherence, respectively, during 48 h of incubation with either the free drugs or the drugs encapsulated in PM (PM/5-FU or PM/PTX). In both cell lines, PM-CON:SMC2 did not show cytotoxic activity at 48 h.

Despite drug efficacy that was observed, no significant differences were detected between free and encapsulated drugs, with or without Ab-SMC2 in both cell lines cultured in adherent conditions. On the other hand, when treatments were applied against tumorsphere formation, remarkable cytotoxic activity was detected with PM-CON:SMC2 in both cell lines. Interestingly, significant stronger activities were also detected for 5-FU and PTX when encapsulated alone or in combination with SMC2, compared to free drugs (Figure 6c). In fact, no efficacy was detected for PTX in MDA-MB-231 (Figure 6c, right panel). These results indicate that encapsulation of 5-FU and PTX into PM improves their performance, in particular when added to CSC-like cells. Of note, the combination of a classical chemotherapeutic agent with Ab-SMC2 was non-deleterious for both encapsulated 5-FU and PTX formulations offering the possibility of eliminating both subpopulations simultaneously, CSC and “bulk” or differentiated cancer cells.

## 4. Discussion

SMC2 forms part of the condensin I and II complexes, thus being a crucial player in several cellular biological processes related to mitotic and meiotic chromosome condensation and rigidity, interphase ribosomal DNA compactation, as well as removal of cohesion during mitosis and meiosis [15,16,17]. Moreover, SMC2 overexpression and mutations in some of the condensing subunits had been reported in cancer genomes, suggesting that functional alterations affecting condensin complexes are common in tumorigenesis [19,20]. Given the essential role of SMC2 in the survival of embryonic stem cells, it is reasonable to speculate that SMC2 could play an important function also in the homeostasis of tumoral CSC. This is a relevant issue since finding an efficient target against this subpopulation is of major importance in order to avoid cancer resistance and tumor recurrence [11,13]. Ideally, a therapy should be able to eradicate not only the primary tumor but also CSC that often survive after most conventional treatments. In order to investigate CSC response to new therapeutic strategies, several methods have been described to generate CSC models in vitro [31]. A simple and rapid approach is based on culturing tumor cell lines in non-adherent conditions, exploiting CSC ability to form pseudo-spherical colonies. This method was firstly reported in 1992 by Reynolds and colleagues [32] and lately improved by Tatianna Herheliuk et al., in 2019 [33], among others.

Aiming to validate SMC2 as a CSC target, SMC2 was silenced in different cancer cell lines (HT116, MDA-MB-231 and PANC-1, from colorectal, breast and pancreatic origin) using RNA interference technology. Its effect was assessed in terms of cell viability and colony formation impairment. As predicted, the silencing of SMC2 was able to reduce cell viability in adherent conditions and the formation of spheres for HCT116 cells in non-adherent cultures. In the case of MDA-MB-231 and PANC-1 the effect of SMC2 silencing was only observed in low attachment conditions. This result revealed that SMC2 could be an important target within stem cell subpopulations (Figure 1 and Figure S1). After SMC2 validation as a promising target, different therapeutic silencing strategies were considered. SMC2 silencing by siRNA arose as the most straightforward approach since a previous report by our group showed effective in vivo tumor growth inhibition after ex vivo silencing of the SMC2 gene in tumor cells [18]. However, unforeseen challenges related with siRNA stability and the need to reach high doses to render biological efficacy after i.v. administration, prompted us to choose an alternative strategy [34]. Thus, we attempted to block SMC2 activity by specific interaction with an antibody against the protein. In order to protect the antibody integrity and improve its intracellular delivery, Ab-SMC2 was conjugated with Pluronic^®^ F127-based PM. The use of nanocarriers allows (i) the systemic delivery of high amount of Ab, (ii) to decrease off-target related toxicity in other organs, (iii) to protect the cargo from enzymatic degradation, and (iv) the sustained release of the Ab alone or in combination with other chemo-therapeutic compounds [9]. In the last years, several formulations have been developed for the intracellular release of antibodies. One of them consists of cationic lipid-based carriers, which are also employed for siRNA delivery, as proven by Courtete et al. (2007) [35]. Nonetheless, even though some of these formulations are reaching clinical trials, strong concerns about their reported toxicity are delaying its entrance into the clinical practice. Moreover, inorganic and viral carriers have also been developed but with the same toxicity and immunogenicity concerns that reduce their clinical expectations. Some polymeric-based nanoparticles also has been developed for intracellular delivery such as the polymersomes described by Canton et al. (2013) and Tian et al. (2014) [36,37], or the self-assembling pyridylthiourea modified polyethylenimine nanoparticles designed by Postupalenko et al. (2014) [8]. Although great efforts have been invested until today, the proposed formulations still present important limitations, such as (i) difficult and expensive production methods, (ii) high mean diameter and immunogenicity issues, (iii) lack of endosomal escape capacity, and (iv) toxicity, among others [9]. In this regard, the proposed delivery system solves some of these drawbacks. Accordingly, they are biocompatible and non-immunogenic, present a small size, and can be produced in compliance to a simple and easily scalable production method. In previous studies, we have demonstrated that these PM can be repeatedly administered in vivo without causing toxicities and can accumulate into tumors upon i.v. administration [38]. Furthermore, our PM were previously used to efficiently conjugate Cetuximab, showing an effective active targeting against the epidermal growth factor receptor (EGFR) in overexpressing breast cancer cells [25].

In the present work, we focused into the intracellular delivery of Ab-SMC2 to silence the activity of SMC2 in CSC. For that, Ab-SMC2 was conjugated to PM through two different approaches. The first one consisted in the encapsulation of the antibody into the micelles hydrophilic shield (PM:SMC2). The second one was based on the covalent bonding of the amine groups of the antibody with the carboxylic terminals of the modified polymer (PM-CON:SMC2). As expected, the formulations with Ab-SMC2 presented larger mean diameter. The conjugation was also confirmed by the FTIR where was possible to see the appearance of a peak at 1646 cm^−1^ (Figure 2 and Figure S3). Since the two encapsulation techniques presented adequate physicochemical features and similar association efficiency, both formulations were tested for cell toxicity in HCT116 and MDA-MB-231 colon and breast cancer cells, to evaluate whether the level of exposure to Ab-SMC2 might affect its in vitro efficacy. As expected, control PM and free antibody did not show cell toxicity in any tested cell lines. However, when conjugated, Ab-SMC2 showed higher efficacy in terms of cell toxicity and impairment of colony formation, suggesting specific positive activity against CSC. In addition, the way Ab-SMC2 was loaded into the PM might affect the efficacy of the formulation, since a slight higher effect was displayed by cells treated with PM-CON:SMC2 in comparison with the ones treated with PM:SMC2 in both tested cell lines (Figure 3). These results and the fact that the covalent conjugation of Ab-SMC2 would be more stable and controlled, made us select PM-CON:SMC2 for further studies. Moreover, the differential effect observed between free antibody and the one conjugated onto PM was clear when analyzing the morphology of treated cells. Thus, it was possible to detect strong morphologic changes in HCT116 cells and the formation of vacuoles in MDA-MB-231 after treatment with PM-CON:SMC2. In contrast, no changes were detected when cells were treated with free antibody or non-conjugated PM. These data indicate that PM-CON:SMC2 were exerting a specific cytotoxic action visibly affecting cellular structures. This was also in accordance with the results seen in the viability assays.

Additionally, in terms of internalization, PM-CON:SMC2 demonstrated to have a faster uptake when compared with the control PM, probably due to its more negatively charged particle surface and Van der Waals interactions between the functional groups of the particle and the cellular membrane. Confocal microscopy images revealed the co-localization of fluorescently labeled PM with endocytic vesicles, suggesting that PM formulations could enter into the cells via endocytosis, at least partially. Other cellular entry routes cannot be discarded, nonetheless. One of the most critical steps regarding the intracellular delivery of antibodies is the need of their endosomal escape. The antibody must be released to the cytosol before reaching the lysosomes, otherwise it might be degraded inside these vehicles. By analyzing the levels of green fluorescence in the whole cytoplasm, it was possible to conclude that a substantial part of Ab-SMC2 PM was able to escape the endosomes and reach the cytoplasm, where the Ab-SMC2 exert its activity. Importantly, these results strongly support the in vitro efficacy outcomes in terms of cell toxicity and colony formation. In order to confirm the biological action of PM-CON:SMC2 inhibiting the SMC2 protein, a cell-cycle assay was performed. Of note, the condensin complex is mostly found in the cell cytoplasm in interphase while during mitosis is found associated to chromatin. Therefore, in order to effectively block SMC2 dimerization and the activity of the condensin complex, the antibody must reach the cell cytosol. Our results demonstrate that at least in the case of HCT116 cells a significant arrest of cell cycle in G1 was driven by the incubation with PM-CON:SMC2, reinforcing our previous results showing a decrease in cell viability. These also suggest the capacity of our system to deliver the antibody to the cell cytoplasm. This pattern, however, was not clear for MDA-MB-231 cells. The effect of the SMC2 inhibition in adherent cultures was restricted to the CSC-like subpopulation in this cell line (Figure 1).

Finally, we further investigated the potential use of PM-CON:SMC2 as adjuvant treatment with Standard-of-Care (SoC) drugs. 5-FU and PTX are the SoC therapy for colon and breast cancer, respectively. Therefore, 5-FU and PTX were loaded into the hydrophobic core of PM-CON:SMC2. In a previous study, we showed that Zileuton™, a drug with reported efficacy in breast CSC, presented higher efficacy when encapsulated in similar micelles than its free form [38]. In agreement with this, both 5-FU and PTX also presented higher efficacy in terms of inhibition of colony formation when encapsulated into PM-CON:SMC2. Interestingly, both 5-FU and PTX showed increased efficacy when encapsulated into PM, particularly regarding tumorsphere formation. This was remarkable in the case of the MDA-MB-231 cell line, known for its high resistance to most treatments. In this case, no efficacy was detected with free PTX (Figure 6c). Although, we could not detect a clear additive efficacy when treating cells with PM-CON:SMC2 loaded with the respective chemotherapeutic drugs, a significant effect was observed in CSC-like in vitro models in comparison with SoC. Our results suggest that the combination of drugs and Ab-SMC2 delivery might cooperate in the eradication not only of bulk tumor cells but also of cells with stemness properties.

## 5. Conclusions

SMC2 block emerges as a promise strategy to complement the current therapies based on chemotherapeutic drugs, intending not only to attack bulk tumor cells but also the remaining CSC. The intracellular delivery of antibodies is still considered a challenge; thus, the discovery of a delivery system with this capacity is of major importance. When conjugated with PM, the Ab-SMC2 is able to reach the cytoplasm, block SMC2 action and obtain a significant impairment in the viability of CSC enriched cultures from colon and breast origin. Moreover, PM were adapted for the combinatory delivery of conventional chemotherapeutic agents and potentially improved their performance. Altogether, the present results suggest that the proposed nanosystem gathers ideal features to ensure the intracellular delivery of Ab-SMC2 into cancer cells. More importantly, it is possible to conclude that by combining PM, drugs and Ab-SMC2, we can make an important contribution, not only to the treatment of the primary tumor, but also in the eradication of the CSC population responsible for tumor recurrence and the metastatic spread of the disease.

## Supplementary Materials

The following are available online at https://www.mdpi.com/1999-4923/12/2/185/s1, Figure S1. SMC2 therapeutic target validation, Figure S2. Stemness gene expression of HCT116 growing in adherent cell cultures or as tumorspheres, Figure S3. FTIR spectra for PM-CON:SMC2 at 100% –COOH, Figure S4. Examples Cell cycle curves fitting.
