# Supplementary material for: Intracellular Delivery of Anti-SMC2 Antibodies against Cancer Stem Cells
Source: Pharmaceutics. 2020 Feb 21;12(2):185. doi: 10.3390/pharmaceutics12020185 (PMC7076674; doi:10.3390/pharmaceutics12020185)
Supplement: Supplementary file 1 [file pharmaceutics-12-00185-s001.pdf]

pharmaceutics

Supplementary Materials: Intracellular Delivery of Anti-SMC2 antibodies Against Cancer Stem Cells

Sara Montero, Joaquin Seras-Franzoso, Fernanda Andrad, Francesc Martinez-Trucharte, Mireia Vilar-Hernández, Manuel Quesada, Helena Xandri, Diego Arango, Ibane Abasolo, Diana Rafael and Simó Schwartz Jr.

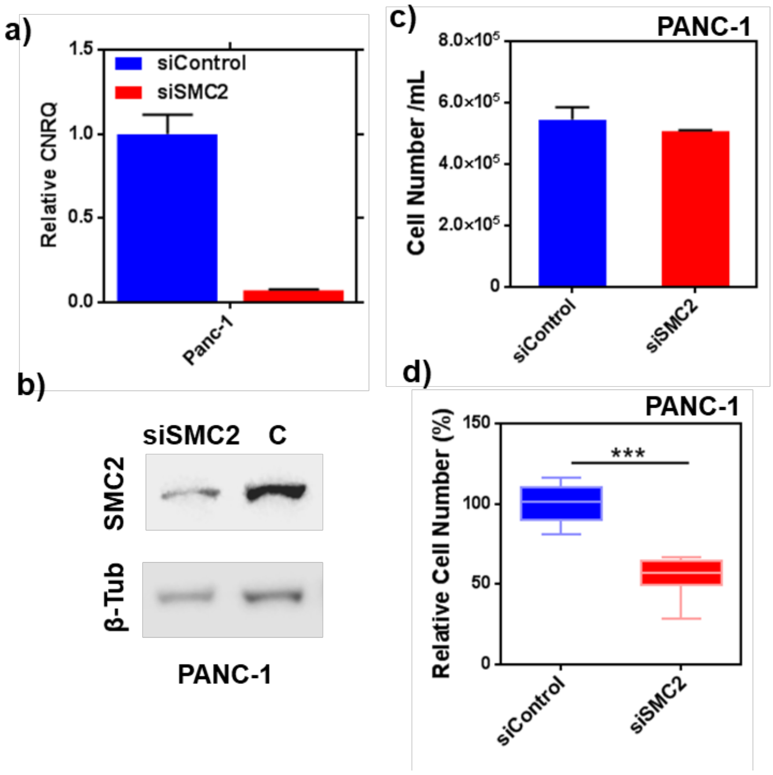

Figure S1. SMC2 therapeutic target validation. a) Relative mRNA levels after SMC2 silencing for PANC-1 pancreatic cancer cell line (siSMC2). Fold change was represented with respect to mRNA levels obtained from non-specific siRNA sequence treated cells (siControl); b) SMC2 and beta-tubulin proteins detected by western blot after SMC2 silencing compared to siControl treated cells (C); c) Cell viability assay for PANC-1 adherent cells after treatment with siSMC2 and siControl; d) Relative cell number percentage for PANC-1 cells growing in non-adherent conditions treated with siSMC2 and siControl.

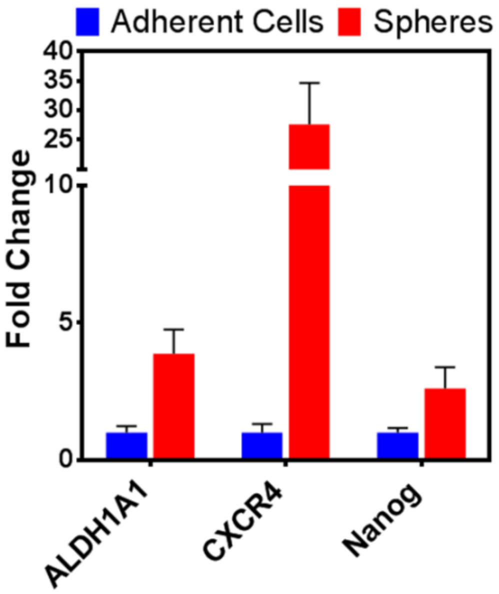

Figure S2. Stemness gene expression of HCT116 cell line growing in adherent cell culture or as tumorspheres (non-attachment). Fold change in gene expression for cells growing as tumorspheres (red bars) are represented with respect to mRNA levels obtained for adherent cells (blue bars). Stemness genes in colon cancer: ALDH1A1, CXCR4 and Nanog were analyzed using GAPDH and β-Actin as housekeeping references.

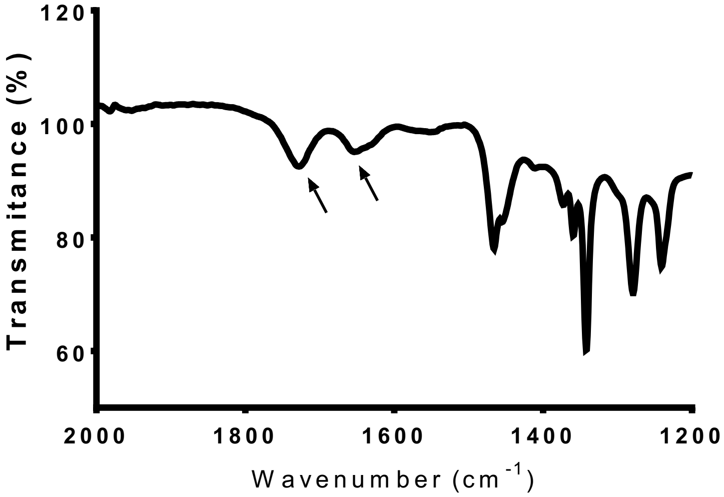

Figure S3: FTIR spectra for PM-CON:SMC2 at 100% -COOH. A peak displayed at 1728 cm^{-1} correspond to the -C=O stretching of the -COOH groups and a peak at 1653 cm^{-1} correspond to the C=O stretching vibrations of the peptide bond of the amide I.

a) HCT116

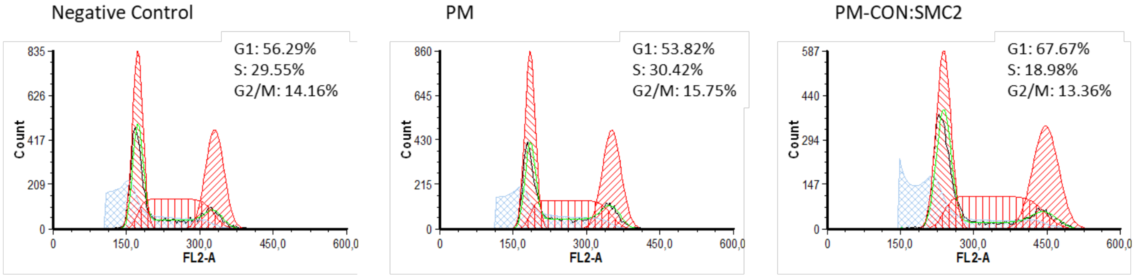

HCT116	% G1 ± SD	% S ± SD	% G2/M ± SD	G2 / G1
**Control**	56.291 ± 2.382	29.546 ± 1.205	14.163 ± 1.223	1.900 ± 0.010
**PM**	53.824 ± 8.322	30.422 ± 7.600	15.754 ± 3.031	1,890 ± 0.031
**PM-CON:SMC2**	**67.667 ± 1.562**	**18.975 ± 1,212**	**13.358 ± 0.364**	**1.876 ± 0.005**

b) MDA-MB.231

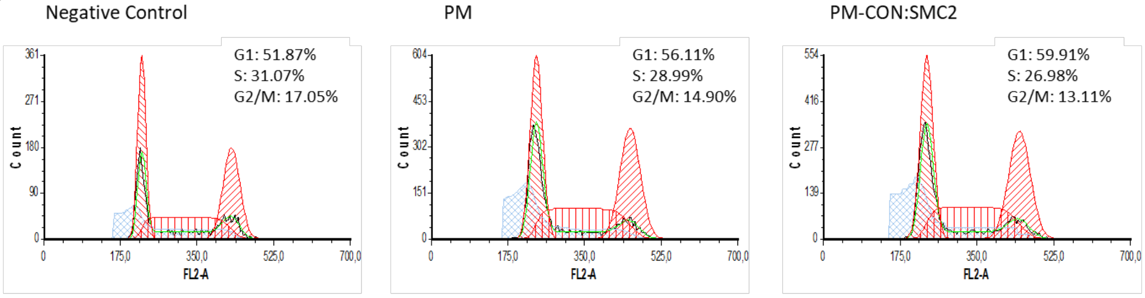

MDA-MB.231	% G1 ± SD	% S ± SD	% G2/M ± SD	G2 / G1
**Control**	51.866 ± 7.764	31.086 ± 5.203	17.047 ± 3.049	1.910 ± 0.009
**PM**	56.111 ± 7.065	28.995 ± 6.389	14.895 ± 0.984	1.902 ± 0.005
**PM-CON:SMC2**	**59.913 ± 6.950**	**26.977 ± 6.519**	**13.110 ± 0.459**	**1.900 ± 0.011**

Figure S4: Representative cell cycle analysis of HCT116 colon cell line (a) and MDA-MB-231 breast cell line (b). Plots of cell cycle fitting curves distribution. Below, quantification of the percentage of cells at distinct cell cycle phases: G1, S and G2/M. G2/G1 ratio was also determined.
